# Who Benefits Most? Interactions between Personality Traits and Outcomes of Four Incremental Meditation and Yoga Treatments

**DOI:** 10.3390/jcm11154553

**Published:** 2022-08-04

**Authors:** Karin Matko, Anne Berghöfer, Michael Jeitler, Peter Sedlmeier, Holger C. Bringmann

**Affiliations:** 1 Institute of Psychology, Chemnitz University of Technology, 09120 Chemnitz, Germany; 2 Institute of Social Medicine, Epidemiology and Health Economics, Charité—Universitätsmedizin Berlin, corporate member of Freie Universität Berlin and Humboldt-Universität zu Berlin, 10117 Berlin, Germany; 3 Department of Psychiatry and Psychotherapy, Krankenhaus Spremberg, 03130 Spremberg, Germany

**Keywords:** mind–body medicine, yoga, meditation, moderators, personality traits, cluster analysis

## Abstract

Mind–Body Medicine (MBM) includes a broad range of interventions with proven preventive and clinical value, such as yoga and meditation. However, people differ in their preferences and response to different MBM treatments and it remains unclear who benefits most from what type of practice. Thus, finding moderators of treatment outcome seems to be a promising approach. This was the aim of the present study. We conducted a single-case multiple-baseline study investigating the outcomes and moderators of four different MBM treatments. Fifty-seven healthy participants with no prior experience were randomly assigned to three baselines (7, 14, and 21 days) and four eight-week treatments: mantra meditation alone, meditation plus physical yoga, meditation plus ethical education and meditation plus yoga and ethical education. We analysed the data using effect size estimation, multiple regression and cluster analyses. High anxiety, high absorption, low spirituality, low openness and younger age were associated with a range of positive outcomes, such as increased wellbeing or decentering and decreased mind wandering. Receiving ethical education consistently improved wellbeing, while engaging in physical yoga reduced mind wandering. In the cluster analysis, we found that participants with a more maladaptive personality structure enhanced their emotion regulation skills more. Consequently, people do differ in their response to MBM interventions and more vulnerable people, or those high in absorption, seem to benefit more. These findings could support the development of custom-tailored MBM interventions and help clinicians to make scientifically sound recommendations for their patients.

## 1. Introduction

Mind–body medicine (MBM), as part of complementary and integrative medicine (CIM), especially yoga and meditation, is becoming increasingly popular in the therapeutic setting, but also as self-medication in prevention and for stress management. These methods are widely used as an adjunct to conventional therapy and evidence of efficacy exists for chronic somatic disorders [1,2], in rehabilitation [3] and in mental disorders [4,5], amongst others. However, it remains unclear who benefits most from what type of practice. Moreover, it is unclear how different components of MBM interventions influence their overall effects. Traditional yoga, for example, entails meditation, postures, breathing practices and ethical guidelines [6,7]. A recent meta-synthesis exemplified how different combinations of these practices have differential and incremental effects on diverse variables and populations [8]. Furthermore, customised treatments were found to be more effective than standard MBM treatments [9,10]. Thus, it seems advisable to identify potential moderators or effective components for specific populations, conditions and individual aims.

Personalised medicine, that is, the treatment of patients based on their individual (phenotypic and (epi-)genetic) predisposition, patient preferences and personality traits, promises to fundamentally transform healthcare. It enables (clinical) research to find specific and more effective strategies for prevention and therapy [11,12]. MBM research so far has shown that people differ in their preferences regarding different psychological treatments [13], meditation techniques [14,15] and practices of multi-component interventions that they use at home [16]. In addition, earlier studies found substantial interindividual variation in response to different contemplative treatments [17,18] and to psychotherapy [19,20]. Several researchers pointed out that personality and other personality traits might tremendously influence the effects of meditation and yoga in this regard [21,22,23]. Yet, only few studies explicitly investigated how different personality traits influence the outcomes of MBM interventions [24,25,26]. Similarly, in MBM research, there is only little research with respect to the questions “which treatment is better for whom, when and why?”—contrary to other areas of psychological and clinical research [27]. Yet, matching individuals by fitting MBM treatments based on their personality and preferences could be valuable. It could reduce the likelihood of attrition, facilitate adherence and self-maintenance of practices and, consequently, improve the effectiveness and outcomes of the intervention [14,23].

Hence, this study investigates which personality traits moderate the outcomes of four different MBM treatments. The treatments are based on the Meditation-Based Lifestyle Modification intervention (MBLM) [28,29], which combines mantra meditation, physical yoga and psychoeducation on the ethical roots of yoga. In this study, we examine four different combinations of these three components using a single-case multiple-baseline design. At the same time, we collected personality traits as possible moderators of treatment outcome. We chose to evaluate a broad range of traits to explore which ones might be of predictive value and to aid future hypothesis generation.

Experimental single-case designs [30] overcome the limitations of conventional group designs, as they take into account heterogeneity between individuals and are, thus, able to depict interindividual changes in response to a treatment. Furthermore, the data are collected continuously over extended periods of time during two phases (baseline and treatment), creating a high time resolution and increasing the reliability of the measurements. Participants in these designs serve as their own control as their baseline data are compared with their treatment data. In multiple-baseline designs, the treatment introduction is staggered across different participants providing experimental control and making horizontal and vertical comparisons possible [31]. Recently, many sophisticated ways of analysing the results of single-case studies have been established, including several suitable effect size measures [32]. Therefore, this design is highly suited to address an explorative research question, such as ours, namely, “Who benefits most from what type of treatment?”. Answering this question might help to make scientifically informed recommendations for different MBM treatments, based on individual characteristics and preferences in the future.

## 2. Materials and Methods

### 2.1. Procedure

The present study represents an additional analysis of data that were previously reported in Matko et al. [17]. It shows an additional aspect of this study by correlating its findings with personality traits. The study used a single-case multiple-baseline design to examine the effects of four different eight-week MBM treatments, based on the MBLM program [28], on a variety of dependent variables. Furthermore, we assessed a multitude of personality traits during pre-test to find possible moderators of the observed effects. Participants received daily online questionnaires throughout their entire baseline and treatment phases. The treatment conditions were mantra meditation alone (MA), mantra meditation plus physical Hatha yoga (MY), mantra meditation plus ethical education (ME) and mantra meditation plus physical yoga and ethical education (MYE). We randomised participants across these four conditions and across three baseline lengths (7, 14 or 21 days) using simple random sampling without replacement. A detailed breakdown of the study methods and the four treatments can be found in Matko et al. [17].

### 2.2. Transparency and Openness

We report our methods, all data exclusions and all measures of the study, and we follow the Single-Case Reporting guideline In BEhavioral interventions [33]. All statistical analyses were performed using R 4.2.0 [34] and the statistical packages *ggpubr* [35], *lm.beta* [36], *rstatix* [37] and *scan* [38]. All scripts and data that support the results can be found at https://osf.io/wjspq/. This study was registered at clinicaltrials.gov under NCT04252976.

### 2.3. Participants

Participants were recruited from the Dresden general community via flyers and mailing lists. They had to be older than 18 years, have no regular yoga or meditation practice and no pre-existing psychiatric conditions or acute psychological issues. To balance class size and expected attrition, we aimed to recruit 12–15 participants per condition. Participation was voluntary and all participants provided written consent to participate in the study. Participants were fully disclosed about the nature of the study prior to randomisation and data collection but could not choose or switch treatments. There was no financial or other compensation for participation in the study, except for the opportunity to win one of ten EUR 50 gift coupons. The institutional review board of the Chemnitz University of Technology approved the experimental protocol.

### 2.4. Treatment

All treatments were jointly led by HCB, an accredited psychiatrist and psychotherapist, and KM, a psychologist and certified yoga instructor with 700 h of teacher training. The length of the weekly sessions varied across conditions, but each condition included a group discussion of experiences at the beginning and end of each session as well as a 25 min silent mantra meditation practice. During the physical Hatha yoga practice, participants learned a set of simple yoga postures, simple breathing techniques, the sun salutation and relaxation techniques. Ethical education followed the protocol developed for the MBLM mind–body program (Bringmann et al., 2020), introducing and discussing a new topic of the yogic *yamas* and *niyamas* each week. We asked all participants to practice their respective treatment practices daily, that is, 20 min of mantra meditation, 20 min of yoga exercises and/or engage in mindful living activities related to the ethical topic of the week.

### 2.5. Measures

As this is one of the first studies addressing the differential effects and moderators of different MBM treatments, our selection of variables was as inclusive as possible. Dependent variables were selected based on theoretical considerations, and a literature search identified relevant personality traits related to meditation. We thoroughly evaluated appropriate measurement instruments through preparatory work and piloting.

All measures were taken online using SoSci Survey [39]. We assessed multiple personality traits during pre-test using various instruments. *Trait absorption* was measured with the 34-item modified German version of the Tellegen Absorption Scale [40]. It reflects a person’s disposition for episodes of fully engaged attention. The 21-item Big Five Inventory—Short Form (BFI-K) [41] was utilised for capturing the *Big Five* personality traits: extraversion, neuroticism, openness to experience, conscientiousness and agreeableness. *Distress tolerance* was assessed using the 15-item Distress Tolerance Scale (DTS) [42]. It measures the degree to which individuals experience negative emotions as intolerable. We employed the 14-item German translation of the Need for Cognition subscale of the Rational–Experiential Inventory [43] to measure participants’ *need for cognition* or, in other words, their engagement in a rational processing style. *Self-compassion* was measured with the 26-item German version of the Self-Compassion Scale (SCS-D) [44], which assesses one’s ability to be kind and forgiving to oneself in difficult circumstances. The 20-item Aspects of Spirituality Questionnaire (ASP-20) [45] was employed to assess participants’ *spirituality*. We measured *trait anxiety* (participants’ general tendency to worry) with the 20-item State–Trait Anxiety Inventory (STAI) [46]. Participants’ *life satisfaction* was measured with the 5-item German version of the Satisfaction with Life Scale (SWLS) [47].

In addition, we assessed the manifestation of the three *gunas* and the *proportion of sattva guna* in our participants using a 79-item version of the Tri-Guna Scales (TGS) [48]. According to the *tri-guna* concept from ancient Indian *Samkhya* philosophy, eudaimonic wellbeing is influenced by three different qualities: the three *gunas sattva* (purity), *rajas* (energy) and *tamas* (inertia). The combination of these three qualities determines the personality of a given person [49].

Over the course of the baseline and treatment phases, we continuously collected several dependent variables: *Affective experience* (valence and arousal) was measured daily with the economic single-item measure Affective Grid [50]. The multi-layered construct of *body awareness* was assessed twice weekly with the help of a newly developed questionnaire combining the 11 items of established questionnaires that were most suited for repeated measurements (see [51]). *Decentering*, which implies having a distanced perspective on one’s internal experiences, was measured weekly with the 11-item Experiences Questionnaire—Decentering Scale (EQ-D) [52]. *Emotion regulation* was measured weekly with a shortened version (see [51]) of the Difficulties in Emotion Regulation Scale (DERS) [53]. *Mind-wandering* was assessed twice weekly using the short, five-item Mind-Wandering Questionnaire (MWQ) [54]. The Result-oriented Problem- and Self-reflection Scale was developed for evaluating coaching outcomes related to increased abilities of self-reflection and self-regulation (RoPS) [55]. We used the subscale Reflection of Concrete Changes from this inventory to assess *self-reflection* once a week. The Perceived Stress Scale (PSS-10) [56] was employed weekly to measure participants’ *stress*. We measured participants’ *wellbeing* daily using the 5-item World Health Organization Wellbeing Index (WHO-5) [57]. All questionnaires (except life satisfaction and the affective grid) were rated on a 5-point Likert scale.

To measure *sustained attention* and vigilance, we employed the Sustained Attention to Response Task (SART) [58] twice weekly. It is a classic go/no-go task. For each trial, participants were presented with a digit from 1 to 9 for 250 ms which was then masked for 900 ms. They were requested to press the space key on each trial (go condition) except for those trials presenting the digit 3 (no-go condition). Altogether there were 207 trials (11.1% no-go, 88.9% go) and the total duration of the task was approximately 5 min. We used the Gorilla Experiment Builder (www.gorilla.sc (accessed on 20 March 2019)) to create and host this task [59]. For this study, we only report changes in the number of incorrect no-go trials (falsely pressing instead of inhibiting the response).

We inverted the effect sizes for attention (*number of incorrect no-go trials*) and emotion regulation (*difficulties*) to increase readability. Thus, higher scores reflect positive increases uniformly in all variables.

### 2.6. Data Analysis

Single-case data can be analysed in multiple ways [60,61] as we reported and outlined in previous publications [17,51]. In the present study, we focus on the effect sizes that we estimated for each dependent variable to assess the treatment effect. We employed Tau-*U*, which is a non-parametric effect size estimate specifically developed for experimental single-case research that allows for controlling trends observed in both phases [62]. We corrected trends in any phase when they were statistically significant or larger than 0.40. An effect size of less than 0.28 indicated a small effect; 0.29–0.47 a moderate effect; 0.48–0.57 a large effect; and 0.58 or above a very large effect [63]. Furthermore, we calculated the *total effect* for each participant. First, we inverted all effect sizes where a negative effect size represented an effect in the expected direction, that is, mind wandering and stress. Then we obtained the average value across all dependent variables.

We conducted multiple regression analyses to determine moderating factors that influenced the observed changes in each dependent variable and the total effect. Therefore, we entered the Tau-*U* effect sizes as criterion and all personality traits as predictors into the regression model. Moreover, we statistically controlled individual practice time, age, gender, occupation and baseline length by including them in the model. Individual practice time represents sum scores of the reported length of each practice participants engaged with at home. In addition, we examined the incremental effects of the four conditions by entering two dummy variables into the model that coded the inclusion of either the physical yoga or the ethical education component. For these two dummy variables, we applied one-tailed tests of significance as we expected combined interventions to have stronger effects than the simple meditation intervention.

We performed cluster analysis to identify groups of participants that were similar in their personality profile and explore how these groups generally responded to the treatments. We first standardised all variables, then calculated Euclidean distances between them and submitted these to a Ward’s hierarchical agglomerative cluster analysis. Then, we inspected the resulting dendrogram to identify the number of clusters present in the data. Next, we exploratively examined differences between the clusters by generating comparative box plots and conducting multiple *t*-tests. We did not correct for multiple comparisons because of the exploratory nature of our analysis. Thereby, we compared clusters regarding the different dependent variables and with respect to the above-mentioned personality traits. In addition, we investigated how the clusters were distributed across the different conditions.

## 3. Results

Fifty-seven participants from the Dresden general community were randomised in this study and 42 participants, 83% women, *M* (*SD*)_age_ = 26.62 (8.37), 86% students and 14% employees, completed the treatment. Of these, 10 participants each completed the MA and MY conditions and 11 participants each the ME and MYE conditions. Sociodemographic data differed slightly across conditions and were, therefore, statistically controlled. Table 1 depicts baseline values for all personality traits as well as comparative values reported in the original studies of the respective scales (if available). We rescaled some of the comparative values to fit the values used in this study, for example, divided sum scores to get mean scores or rescaled 7-point scales to 5-point scales.

The values in Table 1 suggest that our sample was comparable to the original samples of the different scales, except that our participants might have had slightly higher levels of absorption, anxiety and neuroticism.

### 3.1. Moderators of Outcome Variables

Table 2 displays the standardised regression coefficients of each predictor variable on each dependent variable and includes the degrees of freedom and amount of explained variance in each model. All effect size estimates for each dependent variable and full regression tables can be found in the Supplementary Material (Tables S1–S12). There were some missing effect sizes for attention (8), emotion regulation (3) and self-reflection (1) due to missing values in the baseline phase of the respective participants. These were excluded from the following analyses. Regression coefficients that were significant at α < 0.05 were printed in bold, those significant at α < 0.10 were printed in italics.

According to Table 2, *arousal* increased over time for participants high in extraversion and decreased for those high in life satisfaction at baseline. For *decentering*, low openness to experiences predicted greater gains over time, as did high trait anxiety and high sattva proportion at baseline. Moreover, being younger was associated with enhanced decentering. High absorption predicted decreases in *mind wandering*, as did receiving physical yoga. Interestingly, a longer baseline and high spirituality predicted increased mind wandering. Negative changes in *wellbeing* were significantly predicted by age, spirituality and total practice time, suggesting that participants who were either younger, low in spirituality or who practiced less (when everything else was statistically controlled) improved their wellbeing more. Further, participants high in absorption and those who received ethical education in their treatment reported higher gains in wellbeing.

There were no significant predictors of changes in *attention*, *body awareness*, *emotion regulation*, *self-reflection*, *stress* or *valence*. However, as our analysis is exploratory in nature, we also inspected effect sizes that were relatively large and significant on a 10% level. In this respect, participants high in agreeableness and low in neuroticism and openness improved their *attention* skills more. Similarly, *arousal* decreased more for participants who were younger, high in need for cognition or low in sattva proportion. High absorption might be a potentially meaningful predictor of enhanced *body awareness*. Other potentially relevant predictors of enhanced *decentering* skills were high absorption, low spirituality, low conscientiousness and being female. High trait anxiety and low self-compassion predicted improvements in *emotion regulation*. Low agreeableness and receiving ethical education were associated with higher *valence*. Finally, we examined whether personality could predict the *total effect* of the intervention but found no significant predictors. The total variance explained by each model ranged from 0.20 (stress) to 0.72 (emotion regulation).

In sum, high absorption, high anxiety, low spirituality, low openness and younger age seemed to be positive predictors of multiple outcome variables. Regarding the treatment components, ethical education reliably predicted enhanced wellbeing while physical yoga led to reduced mind wandering.

### 3.2. Cluster Analysis

**Figure 1:**
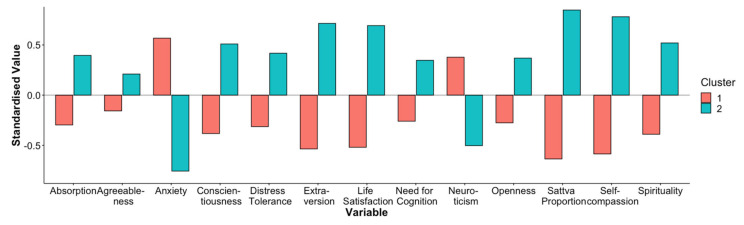
Comparative bar chart displaying the mean of various personality traits for both clusters.

The analysis of the cluster analytic dendrogram (see Supplementary Material Figure S1) suggested a two-cluster solution with 24 participants in cluster 1 and 18 participants in cluster 2. Next, we explored the differences between clusters by plotting the means of all standardised personality traits in both clusters in a comparative bar chart (Figure 1).

Participants in the first cluster exhibited a rather maladaptive personality structure, with higher levels of anxiety and neuroticism and lower levels of distress tolerance, life satisfaction, sattva proportion and self-compassion. They also had lower levels of absorption, extraversion, openness, need for cognition and spirituality. All reported differences were significant at α < 0.05 or lower (see Supplementary Material Table S13). There were no significant differences regarding agreeableness. When we compared the number of participants in each cluster with respect to treatment condition, we found no significant differences, *X*^2^ (3, *N* = 42) = 1.35, *p* = 0.717. However, in the mantra-meditation-alone condition, seven participants were classified as belonging to cluster 1, compared to only three participants in cluster 2.

**Figure 2:**
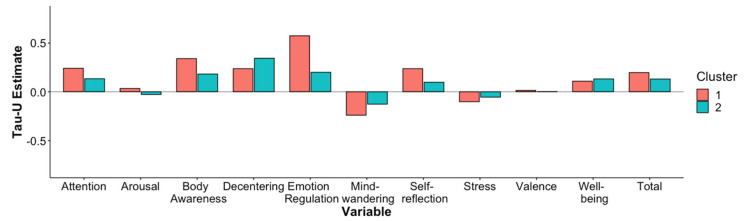
Comparative bar chart displaying the mean of the dependent variables for both clusters.

There were no significant differences in response to the treatments for most dependent variables (Figure 2 and Supplementary Material Table S14). Yet, participants with a more maladaptive personality structure (cluster 1) showed greater increases in emotion regulation than participants with a more balanced personality (cluster 2), *t* (29.9) = 3.61, *p* = 0.001, *r* = 0.36.

## 4. Discussion

This single-case, multiple-baseline study examined the effects of four different eight-week MBM interventions, based on MBLM, on a number of dependent variables, to answer the question of who benefits most from what type of treatment. Thereby, we investigated which personality traits may moderate and predict the outcomes of these four MBM treatments to generate according hypotheses for future research. While high anxiety, high absorption, low spirituality, low openness and younger age predicted stronger effects in a range of positive outcomes, other traits had a more differential impact. Likewise, effects differed depending on the treatment components participants completed. Receiving ethical education improved wellbeing and engaging in physical yoga reduced mind wandering, suggesting possible indications for future research and practice. Similarly, in the cluster analysis, we found that participants with a more maladaptive personality structure (among others: higher anxiety and lower life satisfaction) enhanced their emotion regulation skills more. These results allowed us to address the question of “who benefits most?” from three different perspectives.

### 4.1. Certain Personality Traits Predict Responses to MBM Treatments

High trait absorption facilitated a range of beneficial effects. Participants high in this trait at the onset of the study were more likely to report increased wellbeing, body awareness and decentering as well as decreased mind wandering. Several studies indicate that high trait absorption is related to both greater meditation depth [66] and intense spiritual experiences [67]. Likewise, experienced meditators score higher on trait absorption [68]. However, it remains unclear whether this is the result of self-selection or whether practice of continuous meditation enhanced this trait, or both. In our study, absorption correlated with reported mean meditation ease (*r* = 0.38) and mean meditation duration (*r* = 0.50). This implies that participants high in absorption meditated more frequently and with more effortlessness, which might have increased motivation and effectivity.

Surprisingly, participants low in openness or spirituality experienced more positive effects, such as increased decentering or increased wellbeing and decreased mind wandering, respectively. In contrast, in earlier studies, higher openness was associated with better outcomes in MBM interventions [69] and greater engagement in MBM activities at home [70]. Likewise, spirituality or spiritual experiences moderated positive MBM intervention outcomes [71,72], and openness and spirituality were positively related to psychological wellbeing [73]. Thus, our findings seem somewhat counter intuitive. One explanation might be a ceiling effect, where there might have been little room for improvement for those already very high in those traits. This might also explain why participants high in anxiety benefitted more regarding their decentering abilities or why those low in life satisfaction exhibited a greater reduction in arousal. Furthermore, this corresponds with the results of our cluster analysis (see discussion below). However, there might also be other possible explanations for these findings that we are currently unaware of and that require further investigation.

Younger age was associated with higher decentering and wellbeing over the course of the treatment, the former also being true for participants high in sattva (i.e., a more balanced personality). These factors might have made it easier to accommodate changes throughout the intervention and establish a practice routine. A large survey study reported that middle-aged and older adults had higher levels of decentering than younger ones [74], suggesting younger adults might have more room for improvement in this respect. Interestingly, participants who practiced less enhanced their wellbeing more, supporting a previously reported lack of significant dose–response relationships in mindfulness-based programs [75]. Surprisingly, a longer baseline period predicted higher mind wandering. This might have been due to the extensive study period or might represent a methodological artefact. Moreover, high extraversion led to increased arousal over time. There have been mixed findings regarding the moderating role of extraversion on MBM intervention outcomes [25,76]. Extraversion is robustly associated with positive affect; however, Smillie et al. [77] showed that this positive affect could best be conceptualised as a combination of positive valence and high activation/arousal. Hence, an increase in arousal might essentially represent a positive effect for extraverted participants.

None of our moderators reliably predicted attention, body awareness, emotion regulation, self-reflection, stress or valence. Stress was least explained by our model. This seems surprising considering that earlier studies reported greater declines in stress for participants high in neuroticism or distress tolerance [24,78]. Admittedly, our measure of self-reflection was more specific and less reliable than other measures, which might explain the null findings. The findings for the other variables are harder to judge because previous moderator studies focused primarily on measures of wellbeing or psychopathological symptoms, such as anxiety or depression. However, the non-significant but large effect sizes found in these models indicate possible predictors worth exploring in future studies.

### 4.2. Certain Components of MBM Treatments Can Have Differential Effects and Indications

Ethical education reliably predicted improved wellbeing—even after controlling for a whole range of personality traits. This supports our previously reported findings on the beneficial effects of ethical education [17]. The ethical education component invited participants to explore their personal values and habits, nudging them to make adaptive choices and changes in personal lifestyle. Acting in accordance with personal values has positive effects on wellbeing and quality of life [79]. Furthermore, it is increasingly being acknowledged in psychotherapy and psychiatry, for example, in acceptance and commitment therapy [80]. In addition, this component included more sharing and discussion between participants than the other groups. During these discussions, the group leaders listened nonjudgmentally with acceptance and gave compassionate, supportive responses, thus, modelling the ethical principles taught. These two factors presumably provided social connectedness, which contributed to the participants’ growing feelings of safety, acceptance and openness, and possibly, their greater sense of wellbeing.

Physical yoga, on the other hand, led to reduced mind wandering. Contemporary yoga theories often attribute a reduction in mind wandering to the meditation component of traditional yoga [22,81]. However, in our study, participants in all conditions practiced meditation and only the added physical yoga component markedly decreased mind wandering. Surprisingly, there is little research on physical yoga’s impact on mind wandering, in contrast to meditation research [82]. A recent study indicated that physical yoga reduced mind wandering and this effect was amplified by additional yoga breathing practice [83]. The physical yoga component in this study integrated yoga postures and simple breathing practice, which might have enhanced its effects. In addition, mind wandering and mindfulness were shown to be inversely related, representing opposite sides of the same coin [84]. Fittingly, increased mindfulness has been proposed as a central mechanism of yoga’s effectiveness on stress and general health [85,86].

These findings contribute to a small but growing body of observations that could be used in the future to develop guidelines for personalised MBM recommendations. Physicians expressed a high interest in implementing and recommending MBM interventions, but reported sizeable barriers, such as lack of training, expertise or clinic time [87]. Excessive mind wandering, for example, has been linked to several psychological disorders, such as depression or anxiety [88]. Our findings indicate that a combination of yoga postures, breathing practice and meditation might be particularly helpful in these circumstances. People or patients interested in enhancing their wellbeing, on the other hand, could be advised to practice meditation and engage in ethical education.

### 4.3. Vulnerable Populations Might Benefit More Than Healthy Populations

Our results imply a specifically large increase in emotion regulation skills for those participants with a less favourable personality profile. Emerging evidence suggests that individuals who are more at risk benefit more from emotion regulation interventions [89]. Furthermore, a recent study found that a mindfulness intervention was more effective for those patients with an earlier onset of depression, higher levels of rumination and a lower quality of life [90]. Similar results were obtained for healthy participants with higher levels of neuroticism [24,25] or stress [91]. Hence, it seems that vulnerable participants might benefit more from diverse MBM interventions. Interestingly, this pattern was found in each MBM treatment. In an earlier publication, we reported similar positive effects for all four MBM treatments on emotion regulation [51]. Although almost all participants improved their emotion regulation skills, those with higher anxiety and neuroticism and lower life satisfaction and self-compassion improved more. The reasons for this might be a stronger motivation or a higher potential for improvement. Another potential explanation could be a regression to the mean or a spontaneous remission for those participants that experienced more difficulties. However, this might be unlikely, as in outpatients with clinical depression, MBLM turned out to be more effective than treatment as usual [92].

From a clinical perspective, this might imply that MBM treatments should be recommended for those seeking to enhance their emotion regulatory skills, especially those with more maladaptive traits. Given that emotion regulation difficulties are at the core of various forms of psychopathology [93,94], our findings might be of particular importance in this regard. Previous studies substantiated that diverse MBM treatments enhance emotion regulation, specifically, more adaptive forms like cognitive reappraisal [76,95,96]. In addition, several researchers proposed that MBM practices might train another mindful form of emotion regulation that is associated with interoceptive awareness and non-reactively observing all emotions [97,98,99].

### 4.4. Limitations

This study provides interesting and multi-layered insights into the question “Who benefits most from what type of MBM treatment?”. It employed a rigorous experimental single-case study design, but, nevertheless, has some limitations. In single-case research, a sample of 42 participants is considered exceptionally large. However, the sample size is somewhat small regarding personality effects and subgroup comparisons. Therefore, we adapted the four conditions into larger analysis units and performed regression and cluster analysis across all participants. With up to 85 measurements per participant for daily measures, the effect size estimates of the dependent variables should be very robust. Still, personality research usually acquires much larger samples to rule out incidental findings and increase reliability. If one only looks at the significant results of the regression analyses, one might argue that their number is not much higher than the one to be expected by a Type-1 error. However, due to the relatively small sample size, only relatively large effects reached the 5% limit. Therefore, we reported some non-significant, but relatively high, betas in our regression analyses, which might reach significance in larger samples. It would, thus, be advisable to repeat this study with a conventional design and a larger sample size. Another limitation concerns the shortened and adapted questionnaires that we used to measure body awareness and emotion regulation. The adaptations were necessary to reduce the daily response burden on our participants. Although these measures proved valuable in our study and the extensive piloting we performed, a thorough validation is required.

### 4.5. Future Directions

This study explored a wide range of dependent and independent variables. Its results point to potentially relevant variables and traits that could be of discriminative and predictive value in future research and practice. Future studies should further evaluate the component-specific effects of mind–body interventions. Thereby, the conditions could be adjusted to create comparable session lengths or dismantle the effects of MBM components in an even more detailed way. This could mean comparing treatments incorporating only ethics, only physical postures, only breathing techniques or only meditation to diverse combinations of these. Furthermore, future studies could assess relevant personality traits, such as absorption and anxiety, to examine their moderating influence on treatment outcome. In addition, it would be interesting to compare the responses of healthy participants to those of at-risk or clinical populations. This could be done to further investigate the notion that more vulnerable participants exhibit stronger effects. Moreover, future studies should include measures of motivation and individual preference, as these can strongly impact a treatment’s outcome [100]. Likewise, they could conduct qualitative interviews regarding the experiences of the individual participant to better understand how individuals respond to MBM treatments.

While patient preference is at the forefront of the decision to carry out yoga or not, individualised selection and adaptation to symptomatology and personality traits has hardly been done so far and the evidence is insufficient. The very heterogeneous and contradictory results are possibly due to the so-far undifferentiated indication. A look at the more recent developments in psychotherapy shows that here, too, against the background of high proportions of non-responders and a stagnation of effectiveness, despite constant further developments, a shift to an individualised indication based on biopsychosocial patient characteristics is called for [101]. Further, in the application of complementary medicine, the identification of traits that better predict a therapy response and allow a targeted indication is necessary. Our study contributes to this and could guide future studies examining an individualised prescription of yoga and meditation. Overall, our results showed that participants differ in their response to MBM interventions and that the personality structure and desired skills/goals of people interested in MBM practices can be taken into account. This could support the development of tailored MBM interventions and, in the future, help clinicians to make scientifically sound recommendations for their patients in terms of personalised medicine for optimal healthcare.

## Figures and Tables

**Table 1:** Baseline and comparative values (if available) for all personality traits.

	Our Sample		Comparative Sample		
Variable	*M*	*SD*	*M*	*SD*	Source
Absorption	3.20	0.67	2.92	0.64	Jamieson, 2005 [64]
Agreeableness	3.11	0.79	3.02	0.73	Rammstedt & John, 2005 (Study 1) [41]
Anxiety	2.69	0.67	2.05	0.47	Laux et al., 1981 [46]
Conscientiousness	3.57	0.58	3.53	0.69	Rammstedt & John, 2005 (Study 1) [41]
Distress tolerance	3.41	0.73	3.43	0.76	Simons & Gaher, 2005 [42]
Extraversion	3.24	1.03	3.48	0.87	Rammstedt & John, 2005 (Study 1) [41]
Life satisfaction	4.89	1.22	4.98	1.25	Glaesmer et al., 2011 [47]
Need for cognition	3.70	0.77	3.76	n.a.	Keller et al., 2000 [43]
Neuroticism	3.22	0.93	2.88	0.77	Rammstedt & John, 2005 (Study 1) [41]
Openness	4.14	0.67	3.96	0.62	Rammstedt & John, 2005 (Study 1) [41]
Sattva proportion	0.40	0.06	n.a.	n.a.	n.a.
Self-compassion	2.99	0.59	3.04	0.63	Neff, 2003 [65]
Spirituality	3.18	0.66	n.a.	n.a.	n.a.

*Note*. n.a. = not available.

**Table 2:** Standardised regression estimates of all predictor variables on all dependent variables.

	Attention	Arousal	Body Awareness	Decentering	Emotion Regulation	Mind-Wandering	Self-Reflection	Stress	Valence	Wellbeing
Ethical education (y/n)	−0.07	−0.17	−0.12	−0.21	0.15	−0.01	0.12	−0.02	*0.40* °	0.61 **
Physical yoga (y/n)	−0.20	−0.12	0.30	0.15	0.11	−0.47 *	−0.11	−0.11	0.05	0.30
Absorption	−0.36	0.18	*0.57* °	*0.42* °	0.19	−0.60 *	−0.01	−0.13	0.02	0.61 *
Agreeableness	*0.61* °	−0.11	−0.21	−0.27	−0.26	0.18	0.16	0.07	*−0.59* °	0.17
Anxiety	1.11	−0.15	−0.09	0.92 *	*0.72* °	−0.08	0.21	−0.06	0.40	−0.08
Conscientiousness	−0.80	−0.07	−0.04	*−0.38* °	−0.28	0.15	−0.23	0.27	−0.01	−0.31
Distress tolerance	−0.24	0.15	0.08	0.01	0.18	0.07	−0.01	0.20	0.23	−0.13
Extraversion	−0.41	0.63 *	−0.05	0.05	−0.22	−0.03	0.24	0.33	0.04	−0.25
Life satisfaction	0.41	−0.66 *	−0.23	0.42	0.08	−0.09	0.24	−0.26	0.18	0.30
Need for cognition	0.84	*−0.43* °	0.11	0.01	0.19	−0.24	0.33	−0.18	−0.18	0.28
Neuroticism	*−0.97* °	0.40	0.10	−0.20	0.02	−0.48	−0.06	0.22	0.08	0.41
Openness	*−0.79* °	−0.25	−0.39	−0.54 *	−0.07	0.27	−0.47	0.13	0.29	0.08
Sattva proportion	0.67	*0.74* °	0.54	1.07 **	0.54	−0.35	−0.34	−0.07	0.37	0.47
Self-compassion	−0.41	−0.37	−0.38	0.08	*−0.42* °	0.02	−0.08	0.05	−0.03	−0.07
Spirituality	0.52	−0.46	−0.29	*−0.46* °	−0.41	0.68 *	0.31	0.09	−0.44	−0.83 **
Age	0.37	*−0.47* °	−0.46	−0.46 *	−0.25	0.31	−0.03	0.13	−0.13	−0.51 *
Baseline	0.13	−0.29	0.04	−0.09	−0.30	0.47 *	0.00	−0.01	−0.22	−0.01
Gender (male)	0.03	−0.07	−0.15	−0.37 °	−0.18	0.19	0.03	0.41	−0.20	−0.21
Occupation (employed)	−0.62	0.40	0.46	0.05	−0.09	−0.08	−0.02	0.11	0.04	0.09
Total practice time	−0.11	0.20	0.06	−0.08	0.02	0.15	0.19	−0.08	−0.06	−0.51 *
*df*	13	21	21	21	18	21	20	21	21	21
*R* ^2^	0.53	0.62	0.32	0.70	0.72	0.51	0.44	0.20	0.26	0.60

*Note*. Significance values ** *p* < 0.01, * *p* < 0.05, ° *p* < 0.10.

## Data Availability

Data, analysis and additional materials are openly available at the Open Science Framework (https://osf.io/wjspq/).

## Supplementary Materials

The following supporting information can be downloaded at: https://www.mdpi.com/article/10.3390/jcm11154553/s1. Table S1: Tau-U Effect Size Estimates for Each Participant in All Dependent Variables; Table S2: Full Regression Model for Attention as Dependent Variable and Components, Personality Traits and Sociodemographic Variables as Predictors (df = 13); Table S3: Full Regression Model for Arousal as Dependent Variable and Components, Personality Traits and Sociodemographic Variables as Predictors (df = 21); Table S4: Full Regression Model for Body Awareness as Dependent Variable and Components, Personality Traits and Sociodemographic Variables as Predictors (df = 21); Table S5: Full Regression Model for Decentering as Dependent Variable and Components, Personality Traits and Sociodemographic Variables as Predictors (df = 21); Table S6: Full Regression Model for Emotion Regulation as Dependent Variable and Components, Personality Traits and Sociodemographic Variables as Predictors (df = 18); Table S7: Full Regression Model for Mind-Wandering as Dependent Variable and Components, Personality Traits and Sociodemographic Variables as Predictors (df = 21); Table S8: Full Regression Model for Self-Reflection as Dependent Variable and Components, Personality Traits and Sociodemographic Variables as Predictors (df = 20); Table S9: Full Regression Model for Stress as Dependent Variable and Components, Personality Traits and Sociodemographic Variables as Predictors (df = 21); Table S10: Full Regression Model for Valence as Dependent Variable and Components, Personality Traits and Sociodemographic Variables as Predictors (df = 21); Table S11: Full Regression Model for Well-Being as Dependent Variable and Components, Personality Traits and Sociodemographic Variables as Predictors (df = 21); Table S12: Full Regression Model for the Total Effect as Dependent Variable and Components, Personality Traits and Sociodemographic Variables as Predictors (df = 21); Figure S1: Dendrogram of Relative Distances Between Participants Regarding Their Personality Traits; Table S13: Means, Standard Deviations and t-Test Results Comparing the Manifestation of Personality Traits in Both Clusters (n_1_ = 24, n_2_ = 18); Table S14: Means, Standard Deviations and t-Test Results Comparing the Effects on All Dependent Variables in Both Clusters (n_1_ = 24, n_2_ = 18).
