# Supplementary material for: Who Benefits Most? Interactions between Personality Traits and Outcomes of Four Incremental Meditation and Yoga Treatments
Source: J Clin Med. 2022 Aug 4;11(15):4553. doi: 10.3390/jcm11154553 (PMC9369882; doi:10.3390/jcm11154553)
Supplement: Supplementary file 1 [file jcm-11-04553-s001.zip › jcm-1839369-supplementary.pdf]

Supplementary Material

Who benefits most? Interactions between personality traits and outcomes of four incremental meditation and yoga treatments

Matko, Berghöfer, Jeitler, Sedlmeier, & Bringmann (2022)

Table S1

Tau-U *Effect Size Estimates for Each Participant in All Dependent Variables*

Case	Attention	Arousal	Body awareness	Decentering	Emotion regulation	Mind-wandering	Self-reflection	Stress	Valence	Wellbeing	Total
1	0.30	0.23	0.67	0.47	-0.65	-0.71	-0.02	0.07	0.18	0.25	0.23
2	n.a.	-0.09	0.56	0.74	-0.50	-0.68	0.06	-0.12	-0.20	-0.11	0.27
3	n.a.	0.12	-0.11	0.39	-0.29	0.63	0.37	0.29	-0.09	-0.46	0.00
4	0.12	-0.24	-0.37	0.13	0.41	0.40	0.22	0.27	-0.39	-0.51	-0.19
5	-0.74	0.04	-0.56	0.29	-0.23	-0.48	-0.49	0.37	-0.25	0.24	0.03
6	-0.13	-0.12	0.50	0.58	-0.85	-0.19	0.34	-0.22	0.18	0.13	0.32
7	n.a.	0.05	-0.07	-0.25	n.a.	0.30	-0.54	0.03	-0.21	-0.16	-0.20
8	-0.20	0.10	-0.07	0.07	-0.48	-0.53	0.55	0.30	0.34	0.16	0.18
9	-0.48	0.40	0.62	0.67	-0.80	-0.60	0.66	-0.53	-0.13	0.06	0.39
10	-0.87	0.10	0.70	0.88	0.02	0.07	-0.42	0.59	-0.14	0.07	0.12
11	0.01	0.47	0.63	0.49	0.06	-0.31	0.58	0.38	-0.01	0.21	0.13
12	n.a.	0.23	0.43	0.56	-0.71	-0.03	0.67	-0.39	0.25	0.36	0.35
13	-0.76	0.05	0.70	0.19	-0.52	-0.39	-0.29	0.20	0.37	0.14	0.25
14	n.a.	0.18	0.40	0.47	-0.91	-0.23	0.60	-0.73	0.13	0.45	0.41
15	-0.25	-0.33	0.87	0.89	-0.39	-0.32	0.06	-0.24	0.36	0.46	0.42
16	-0.54	-0.25	0.63	0.29	0.20	0.23	0.61	-0.29	0.08	0.31	0.26
17	-0.24	-0.21	-0.28	0.29	-0.52	0.39	0.66	0.15	-0.19	-0.15	0.08
18	0.45	-0.27	0.26	-0.11	-0.11	-0.13	-0.16	-0.15	0.33	0.36	0.09
19	0.09	0.13	0.67	-0.11	-0.77	0.52	0.68	0.27	-0.22	0.06	0.08
20	0.62	0.11	-0.33	0.00	-0.50	-0.48	0.21	-0.71	0.10	0.14	0.11
21	-0.84	-0.14	0.11	-0.50	-0.67	0.04	n.a.	-0.33	-0.16	0.30	0.19
22	0.22	0.01	0.85	0.91	-0.70	-0.33	-0.06	-0.74	-0.05	0.43	0.36

23	n.a.	0.00	0.51	0.92	-0.82	-0.39	0.87	-0.44	-0.14	0.00	0.42
24	-0.56	-0.07	0.59	-0.26	-0.38	0.31	0.50	0.29	-0.12	-0.14	0.10
25	0.30	-0.12	0.48	0.81	-0.57	-0.18	-0.75	-0.21	-0.08	0.15	0.14
26	-0.54	-0.35	0.67	0.06	-0.71	-0.86	-0.13	-0.69	0.26	0.27	0.43
27	-0.61	-0.26	0.44	0.32	-0.42	-0.33	0.29	-0.39	0.04	0.25	0.34
28	-0.48	0.00	0.81	0.71	n.a.	-0.55	0.57	-0.61	-0.21	0.22	0.42
29	0.29	0.18	-0.58	-0.67	n.a.	-0.60	-0.95	0.40	0.07	-0.14	-0.28
30	n.a.	0.18	0.28	-0.24	0.20	-0.25	0.15	0.35	0.19	-0.14	-0.02
31	-0.56	-0.03	-0.56	0.43	-0.33	0.20	-0.30	-0.41	0.07	0.09	0.09
32	-0.10	0.36	0.55	0.57	-0.50	-0.51	0.72	0.25	-0.19	0.33	0.25
33	0.07	-0.12	0.60	0.68	-0.75	-0.52	0.81	-0.11	0.12	0.19	0.38
34	0.00	-0.12	0.46	-0.67	-0.20	-0.11	0.00	0.83	0.26	0.34	0.00
35	n.a.	-0.27	-0.31	-0.57	-0.70	-0.48	-0.22	0.33	0.16	0.23	0.05
36	n.a.	0.15	0.29	0.54	-0.43	-0.39	0.42	-0.63	-0.03	0.37	0.32
37	0.00	0.21	0.43	0.54	-0.61	-0.67	0.29	-0.25	0.04	0.25	0.29
38	0.16	0.38	0.38	-0.53	0.57	0.32	0.26	0.87	-0.25	-0.30	-0.28
39	-0.30	-0.30	-0.40	0.52	0.08	0.13	0.20	0.09	-0.21	0.24	0.06
40	-0.01	-0.25	-0.59	0.35	-0.32	0.24	-0.40	-0.41	-0.09	-0.09	-0.01
41	-0.09	0.27	0.47	0.36	-0.53	-0.24	0.29	-1.00	0.03	0.19	0.29
42	-0.16	-0.09	0.13	0.69	-0.33	-0.35	0.31	-0.17	0.18	-0.04	0.24

Note. n.a. = not available

Table S2

*Full Regression Model for Attention as Dependent Variable and Components, Personality Traits and Sociodemographic Variables as Predictors (*df *= 13)*

Variable	*b*	β	*SE*	*t*	*p*
(Intercept)	-0.26	0.00	2.13	-0.12	0.906
Ethical education (y/n)	-0.06	-0.07	0.27	-0.21	0.419
Physical yoga (y/n)	-0.16	-0.20	0.29	-0.55	0.297
Absorption	-0.21	-0.36	0.23	-0.93	0.371
Agreeableness	0.30	0.61	0.17	1.82	0.093
Anxiety	0.63	1.11	0.45	1.40	0.186
Conscientiousness	-0.54	-0.80	0.37	-1.45	0.170
Distress tolerance	-0.13	-0.24	0.25	-0.53	0.606
Extraversion	-0.16	-0.41	0.15	-1.10	0.293
Life satisfaction	0.13	0.41	0.17	0.78	0.451
Need for cognition	0.41	0.84	0.24	1.76	0.103
Neuroticism	-0.41	-0.97	0.21	-1.92	0.078
Openness	-0.44	-0.79	0.21	-2.08	0.058
Sattva proportion	4.37	0.67	4.05	1.08	0.300
Self-compassion	-0.25	-0.41	0.22	-1.12	0.283
Spirituality	0.33	0.52	0.23	1.42	0.180
Age	0.02	0.37	0.02	0.80	0.439
Baseline	0.01	0.13	0.02	0.47	0.647
Gender (male)	0.03	0.03	0.37	0.08	0.936
Occupation (employed)	-0.63	-0.62	0.63	-1.01	0.332
Total practice time	0.00	-0.11	0.00	-0.35	0.733

Table S3

*Full Regression Model for Arousal as Dependent Variable and Components, Personality Traits and Sociodemographic Variables as Predictors (*df *= 21)*

Variable	*b*	β	*SE*	*t*	*p*
(Intercept)	0.84	0.00	0.82	1.03	0.316
Ethical education (y/n)	-0.07	-0.17	0.09	-0.84	0.206
Physical yoga (y/n)	-0.05	-0.12	0.09	-0.56	0.292
Absorption	0.06	0.18	0.08	0.77	0.447
Agreeableness	-0.03	-0.11	0.06	-0.50	0.622
Anxiety	-0.05	-0.15	0.14	-0.34	0.739
Conscientiousness	-0.03	-0.07	0.09	-0.30	0.766
Distress tolerance	0.04	0.15	0.08	0.57	0.575
Extraversion	0.13	0.63	0.05	2.59	0.017
Life satisfaction	-0.12	-0.66	0.05	-2.20	0.039
Need for cognition	-0.12	-0.43	0.07	-1.82	0.083
Neuroticism	0.09	0.40	0.07	1.34	0.196
Openness	-0.08	-0.25	0.08	-1.07	0.299
Sattva proportion	2.74	0.74	1.36	2.02	0.057
Self-compassion	-0.14	-0.37	0.08	-1.67	0.110
Spirituality	-0.15	-0.46	0.09	-1.64	0.116
Age	-0.01	-0.47	0.01	-2.04	0.055
Baseline	-0.01	-0.29	0.01	-1.65	0.115
Gender (male)	-0.04	-0.07	0.13	-0.31	0.756
Occupation (employed)	0.25	0.40	0.15	1.63	0.119
Total practice time	0.00	0.20	0.00	0.95	0.354

Table S4

*Full Regression Model for Body Awareness as Dependent Variable and Components, Personality Traits and Sociodemographic Variables as Predictors (*df *= 21)*

Variable	*b*	β	*SE*	*t*	*p*
(Intercept)	0.93	0.00	2.21	0.42	0.679
Ethical education (y/n)	-0.10	-0.12	0.23	-0.43	0.335
Physical yoga (y/n)	0.26	0.30	0.25	1.04	0.152
Absorption	0.38	0.57	0.21	1.83	0.081
Agreeableness	-0.12	-0.21	0.16	-0.74	0.467
Anxiety	-0.06	-0.09	0.39	-0.15	0.881
Conscientiousness	-0.03	-0.04	0.23	-0.13	0.895
Distress tolerance	0.05	0.08	0.21	0.25	0.809
Extraversion	-0.02	-0.05	0.14	-0.14	0.891
Life satisfaction	-0.08	-0.23	0.15	-0.56	0.579
Need for cognition	0.06	0.11	0.18	0.35	0.733
Neuroticism	0.05	0.10	0.19	0.25	0.807
Openness	-0.26	-0.39	0.21	-1.25	0.226
Sattva proportion	4.10	0.54	3.69	1.11	0.279
Self-compassion	-0.28	-0.38	0.22	-1.28	0.214
Spirituality	-0.19	-0.29	0.25	-0.77	0.449
Age	-0.02	-0.46	0.02	-1.51	0.146
Baseline	0.00	0.04	0.02	0.16	0.878
Gender (male)	-0.18	-0.15	0.35	-0.50	0.619
Occupation (employed)	0.57	0.46	0.41	1.40	0.175
Total practice time	0.00	0.06	0.00	0.21	0.837

Table S5

*Full Regression Model for Decentering as Dependent Variable and Components, Personality Traits and Sociodemographic Variables as Predictors (*df *= 21)*

Variable	*b*	β	*SE*	*t*	*p*
(Intercept)	-1.33	0.00	1.51	-0.88	0.387
Ethical education (y/n)	-0.19	-0.21	0.16	-1.19	0.123
Physical yoga (y/n)	0.14	0.15	0.17	0.80	0.228
Absorption	0.29	0.42	0.14	2.01	0.057
Agreeableness	-0.16	-0.27	0.11	-1.44	0.164
Anxiety	0.62	0.92	0.27	2.32	0.031
Conscientiousness	-0.29	-0.38	0.16	-1.86	0.078
Distress tolerance	0.01	0.01	0.14	0.04	0.971
Extraversion	0.02	0.05	0.10	0.23	0.824
Life satisfaction	0.16	0.42	0.10	1.59	0.128
Need for cognition	0.01	0.01	0.12	0.05	0.963
Neuroticism	-0.09	-0.20	0.13	-0.74	0.466
Openness	-0.37	-0.54	0.14	-2.58	0.018
Sattva proportion	8.27	1.07	2.52	3.29	0.004
Self-compassion	0.06	0.08	0.15	0.41	0.688
Spirituality	-0.32	-0.46	0.17	-1.86	0.078
Age	-0.03	-0.46	0.01	-2.28	0.033
Baseline	-0.01	-0.09	0.01	-0.56	0.585
Gender (male)	-0.44	-0.37	0.24	-1.85	0.078
Occupation (employed)	0.06	0.05	0.28	0.21	0.836
Total practice time	0.00	-0.08	0.00	-0.43	0.672

Table S6

*Full Regression Model for Emotion Regulation as Dependent Variable and Components, Personality Traits and Sociodemographic Variables as Predictors (*df *= 18)*

Variable	*b*	β	*SE*	*t*	*p*
(Intercept)	0.30	0.00	1.19	0.25	0.805
Ethical education (y/n)	0.11	0.15	0.13	0.83	0.208
Physical yoga (y/n)	0.08	0.11	0.14	0.56	0.291
Absorption	0.10	0.19	0.12	0.86	0.401
Agreeableness	-0.11	-0.26	0.09	-1.29	0.214
Anxiety	0.39	0.72	0.21	1.85	0.082
Conscientiousness	-0.17	-0.28	0.14	-1.22	0.238
Distress tolerance	0.09	0.18	0.13	0.71	0.490
Extraversion	-0.08	-0.22	0.08	-1.08	0.295
Life satisfaction	0.03	0.08	0.10	0.25	0.804
Need for cognition	0.09	0.19	0.10	0.82	0.422
Neuroticism	0.01	0.02	0.10	0.07	0.948
Openness	-0.04	-0.07	0.12	-0.31	0.760
Sattva proportion	3.21	0.54	2.04	1.58	0.133
Self-compassion	-0.25	-0.42	0.12	-2.05	0.055
Spirituality	-0.22	-0.41	0.14	-1.56	0.136
Age	-0.01	-0.25	0.01	-1.14	0.271
Baseline	-0.02	-0.30	0.01	-1.74	0.099
Gender (male)	-0.16	-0.18	0.19	-0.86	0.403
Occupation (employed)	-0.09	-0.09	0.23	-0.39	0.701
Total practice time	0.00	0.02	0.00	0.08	0.937

Table S7

*Full Regression Model for Mind-Wandering as Dependent Variable and Components, Personality Traits and Sociodemographic Variables as Predictors (*df *= 21)*

Variable	*b*	β	*SE*	*t*	*p*
(Intercept)	-0.28	0.00	1.61	-0.18	0.861
Ethical education (y/n)	-0.01	-0.01	0.17	-0.03	0.487
Physical yoga (y/n)	-0.35	-0.47	0.18	-1.89	0.036
Absorption	-0.34	-0.60	0.15	-2.24	0.036
Agreeableness	0.09	0.18	0.12	0.76	0.455
Anxiety	-0.05	-0.08	0.28	-0.16	0.872
Conscientiousness	0.10	0.15	0.17	0.58	0.566
Distress tolerance	0.03	0.07	0.15	0.23	0.824
Extraversion	-0.01	-0.03	0.10	-0.10	0.924
Life satisfaction	-0.03	-0.09	0.11	-0.28	0.783
Need for cognition	-0.12	-0.24	0.13	-0.90	0.379
Neuroticism	-0.20	-0.48	0.14	-1.44	0.166
Openness	0.15	0.27	0.15	1.00	0.331
Sattva proportion	-2.26	-0.35	2.68	-0.84	0.408
Self-compassion	0.01	0.02	0.16	0.08	0.935
Spirituality	0.39	0.68	0.18	2.15	0.043
Age	0.01	0.31	0.01	1.18	0.252
Baseline	0.03	0.47	0.01	2.33	0.030
Gender (male)	0.19	0.19	0.25	0.74	0.470
Occupation (employed)	-0.09	-0.08	0.30	-0.29	0.774
Total practice time	0.00	0.15	0.00	0.65	0.522

Table S8

*Full Regression Model for Self-Reflection as Dependent Variable and Components, Personality Traits and Sociodemographic Variables as Predictors (*df *= 20)*

Variable	*b*	β	*SE*	*t*	*p*
(Intercept)	0.55	0.00	2.24	0.25	0.807
Ethical education (y/n)	0.10	0.12	0.22	0.47	0.322
Physical yoga (y/n)	-0.10	-0.11	0.24	-0.41	0.345
Absorption	-0.01	-0.01	0.20	-0.05	0.961
Agreeableness	0.09	0.16	0.15	0.58	0.569
Anxiety	0.14	0.21	0.38	0.36	0.719
Conscientiousness	-0.18	-0.23	0.23	-0.79	0.441
Distress tolerance	-0.01	-0.01	0.20	-0.04	0.971
Extraversion	0.10	0.24	0.13	0.76	0.459
Life satisfaction	0.09	0.24	0.14	0.65	0.526
Need for cognition	0.19	0.33	0.17	1.10	0.286
Neuroticism	-0.03	-0.06	0.18	-0.17	0.869
Openness	-0.31	-0.47	0.20	-1.58	0.130
Sattva proportion	-2.64	-0.34	3.96	-0.67	0.512
Self-compassion	-0.06	-0.08	0.21	-0.31	0.764
Spirituality	0.21	0.31	0.24	0.87	0.397
Age	0.00	-0.03	0.02	-0.12	0.907
Baseline	0.00	0.00	0.02	0.02	0.984
Gender (male)	0.04	0.03	0.33	0.12	0.902
Occupation (employed)	-0.02	-0.02	0.38	-0.06	0.953
Total practice time	0.00	0.19	0.00	0.73	0.474

Table S9

*Full Regression Model for Stress as Dependent Variable and Components, Personality Traits and Sociodemographic Variables as Predictors (*df *= 21)*

Variable	*b*	β	*SE*	*t*	*p*
(Intercept)	-1.55	0.00	2.43	-0.64	0.530
Ethical education (y/n)	-0.02	-0.02	0.26	-0.07	0.474
Physical yoga (y/n)	-0.10	-0.11	0.28	-0.36	0.361
Absorption	-0.09	-0.13	0.23	-0.39	0.703
Agreeableness	0.04	0.07	0.18	0.23	0.820
Anxiety	-0.04	-0.06	0.43	-0.09	0.933
Conscientiousness	0.21	0.27	0.26	0.83	0.418
Distress tolerance	0.12	0.20	0.23	0.53	0.599
Extraversion	0.14	0.33	0.15	0.94	0.357
Life satisfaction	-0.09	-0.26	0.16	-0.59	0.559
Need for cognition	-0.10	-0.18	0.20	-0.52	0.612
Neuroticism	0.10	0.22	0.21	0.51	0.617
Openness	0.08	0.13	0.23	0.37	0.717
Sattva proportion	-0.55	-0.07	4.05	-0.14	0.893
Self-compassion	0.04	0.05	0.24	0.16	0.876
Spirituality	0.06	0.09	0.28	0.23	0.819
Age	0.01	0.13	0.02	0.39	0.700
Baseline	0.00	-0.01	0.02	-0.03	0.977
Gender (male)	0.49	0.41	0.38	1.26	0.220
Occupation (employed)	0.13	0.11	0.45	0.30	0.768
Total practice time	0.00	-0.08	0.00	-0.25	0.802

Table S10

Full Regression Model for Valence as Dependent Variable and Components, Personality Traits and Sociodemographic Variables as Predictors (df = 21)

Variable	*b*	β	*SE*	*t*	*p*
(Intercept)	-0.39	0.00	1.02	-0.38	0.709
Ethical education (y/n)	0.15	0.40	0.11	1.43	0.084
Physical yoga (y/n)	0.02	0.05	0.12	0.15	0.440
Absorption	0.01	0.02	0.10	0.06	0.950
Agreeableness	-0.15	-0.59	0.07	-2.01	0.057
Anxiety	0.12	0.40	0.18	0.64	0.527
Conscientiousness	0.00	-0.01	0.11	-0.02	0.984
Distress tolerance	0.06	0.23	0.10	0.66	0.517
Extraversion	0.01	0.04	0.06	0.11	0.912
Life satisfaction	0.03	0.18	0.07	0.44	0.663
Need for cognition	-0.05	-0.18	0.08	-0.56	0.581
Neuroticism	0.02	0.08	0.09	0.20	0.843
Openness	0.09	0.29	0.10	0.88	0.391
Sattva proportion	1.26	0.37	1.71	0.74	0.470
Self-compassion	-0.01	-0.03	0.10	-0.09	0.932
Spirituality	-0.13	-0.44	0.12	-1.14	0.266
Age	0.00	-0.13	0.01	-0.40	0.691
Baseline	-0.01	-0.22	0.01	-0.88	0.389
Gender (male)	-0.10	-0.20	0.16	-0.63	0.536
Occupation (employed)	0.02	0.04	0.19	0.11	0.914
Total practice time	0.00	-0.06	0.00	-0.19	0.849

Table S11

*Full Regression Model for Well-Being as Dependent Variable and Components, Personality Traits and Sociodemographic Variables as Predictors (*df *= 21)*

Variable	*b*	β	*SE*	*t*	*p*
(Intercept)	-0.19	0.00	0.90	-0.21	0.835
Ethical education (y/n)	0.28	0.61	0.09	2.97	0.004
Physical yoga (y/n)	0.14	0.30	0.10	1.32	0.109
Absorption	0.21	0.61	0.08	2.52	0.020
Agreeableness	0.05	0.17	0.06	0.76	0.456
Anxiety	-0.03	-0.08	0.16	-0.19	0.855
Conscientiousness	-0.12	-0.31	0.09	-1.30	0.206
Distress tolerance	-0.04	-0.13	0.08	-0.51	0.615
Extraversion	-0.06	-0.25	0.06	-1.01	0.326
Life satisfaction	0.06	0.30	0.06	0.99	0.336
Need for cognition	0.08	0.28	0.07	1.14	0.266
Neuroticism	0.10	0.41	0.08	1.35	0.190
Openness	0.03	0.08	0.09	0.31	0.757
Sattva proportion	1.86	0.47	1.50	1.24	0.228
Self-compassion	-0.03	-0.07	0.09	-0.30	0.769
Spirituality	-0.29	-0.83	0.10	-2.88	0.009
Age	-0.01	-0.51	0.01	-2.16	0.043
Baseline	0.00	-0.01	0.01	-0.07	0.945
Gender (male)	-0.13	-0.21	0.14	-0.92	0.366
Occupation (employed)	0.06	0.09	0.17	0.37	0.713
Total practice time	0.00	-0.51	0.00	-2.43	0.024

Table S12

*Full Regression Model for the Total Effect as Dependent Variable and Components, Personality Traits and Sociodemographic Variables as Predictors (*df *= 21)*

Variable	*b*	β	*SE*	*t*	*p*
(Intercept)	0.10	0.00	0.85	0.12	0.907
Ethical education (y/n)	0.03	0.08	0.09	0.35	0.366
Physical yoga (y/n)	0.10	0.27	0.10	1.05	0.153
Absorption	0.14	0.47	0.08	1.70	0.104
Agreeableness	-0.03	-0.13	0.06	-0.54	0.598
Anxiety	0.15	0.52	0.15	0.99	0.332
Conscientiousness	-0.14	-0.43	0.09	-1.59	0.127
Distress tolerance	-0.02	-0.08	0.08	-0.27	0.792
Extraversion	-0.03	-0.15	0.05	-0.52	0.608
Life satisfaction	0.08	0.49	0.06	1.39	0.178
Need for cognition	0.10	0.39	0.07	1.39	0.179
Neuroticism	-0.01	-0.05	0.07	-0.15	0.884
Openness	-0.13	-0.44	0.08	-1.58	0.129
Sattva proportion	1.64	0.50	1.41	1.16	0.259
Self-compassion	-0.06	-0.18	0.08	-0.67	0.508
Spirituality	-0.12	-0.41	0.10	-1.23	0.231
Age	-0.01	-0.41	0.01	-1.52	0.145
Baseline	0.00	-0.07	0.01	-0.36	0.725
Gender (male)	-0.16	-0.32	0.13	-1.22	0.237
Occupation (employed)	0.04	0.08	0.16	0.27	0.791
Total practice time	0.00	-0.09	0.00	-0.36	0.721

Figure S1

Dendrogram of Relative Distances Between Participants Regarding Their Personality Traits

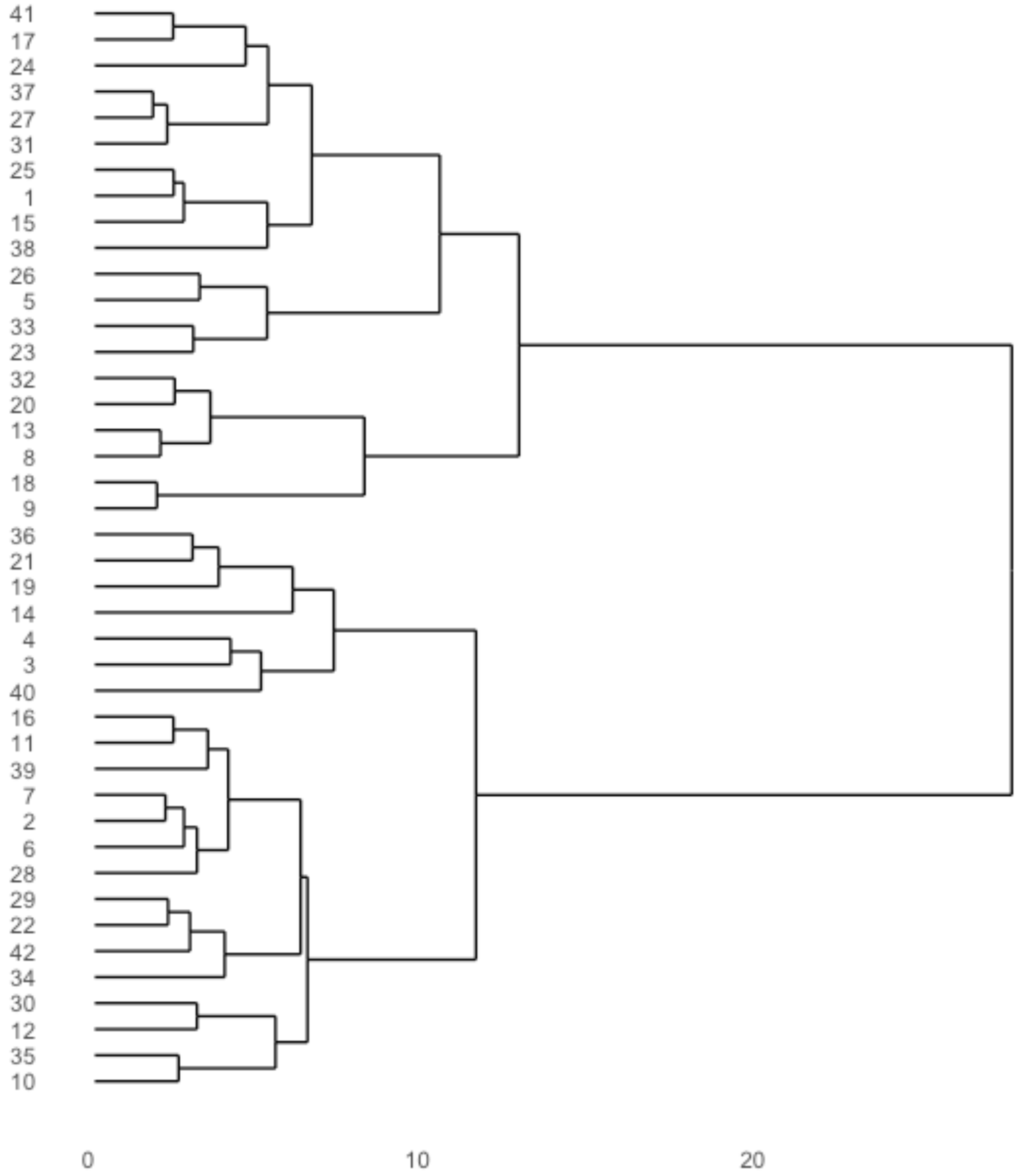

Note. Generated with the R package *ggdendro* (de Vries & Ripley, 2020).

Table S13

Means, Standard Deviations and t*-Test Results Comparing the Manifestation of Personality Traits in Both Clusters (*$n_1 = 24$*,* $n_2 = 18$*)*

Variable	*M*	*SD*	*t*	*df*	*p*	*r*
Absorption	3.00	0.62	-2.32	35.6	0.026	0.13
	3.46	0.65				
Agreeableness	2.99	0.80	-1.19	38.0	0.240	0.04
	3.28	0.75				
Anxiety	3.07	0.42	5.32	28.5	0.000	0.50
	2.18	0.61				
Conscientiousness	3.34	0.55	-3.22	38.8	0.003	0.21
	3.86	0.49				
Distress tolerance	3.18	0.64	-2.43	32.8	0.021	0.15
	3.71	0.76				
Extraversion	2.69	0.81	-5.08	36.7	0.000	0.41
	3.97	0.81				
Life satisfaction	4.25	1.17	-5.22	37.6	0.000	0.42
	5.73	0.66				
Need for cognition	3.50	0.83	-2.11	40.0	0.041	0.10
	3.97	0.60				
Neuroticism	3.57	0.76	3.01	31.9	0.005	0.22
	2.75	0.95				
Openness	3.96	0.78	-2.36	35.4	0.024	0.14
	4.39	0.39				
Sattva proportion	0.36	0.03	-6.70	29.2	0.000	0.61
	0.45	0.05				
Self-compassion	2.64	0.41	-5.80	33.3	0.000	0.50
	3.45	0.47				
Spirituality	2.92	0.63	-3.32	39.4	0.002	0.22
	3.52	0.53				

Note. The upper values *(M, SD)* represents the values of Cluster 1, the lower values of Cluster 2.

Table S14

Means, Standard Deviations and t-Test Results Comparing the Effects on All Dependent Variables in Both Clusters (n_1 = 24, n_2 = 18)

Variable	*M*	*SD*	*t*	*df*	*p*	*r*
Attention	0.24	0.35	0.78	28.9	0.442	0.02
	0.13	0.44				
Arousal	0.03	0.19	0.88	31.1	0.384	0.02
	-0.03	0.25				
Body awareness	0.34	0.39	1.11	31.4	0.277	0.04
	0.18	0.50				
Decentering	0.24	0.50	-0.78	40.0	0.437	0.02
	0.34	0.38				
Emotion regulation	0.57	0.18	3.61	22.9	0.001	0.36
	0.20	0.41				
Mind-wandering	-0.24	0.41	-0.99	39.9	0.330	0.02
	-0.13	0.33				
Self-reflection	0.24	0.46	1.00	37.5	0.325	0.03
	0.10	0.43				
Stress	-0.10	0.46	-0.34	37.4	0.738	0.00
	-0.06	0.44				
Valence	0.01	0.20	0.19	36.4	0.847	0.00
	0.00	0.20				
Well-being	0.11	0.22	-0.31	34.0	0.760	0.00
	0.13	0.25				
Total Effect	0.20	0.19	1.11	36.3	0.273	0.03
	0.13	0.19				

Note. The upper values *(M, SD)* represent the values of Cluster 1, the lower values of Cluster 2.
